# Can *Qigong* Be a Tool to Assist Students in Handling COVID-19’s Resulting Academic Stress?

**DOI:** 10.3390/healthcare11030307

**Published:** 2023-01-19

**Authors:** Mário Gonçalves, Leonel Duarte, Jorge Magalhães Rodrigues, Henry Johannes Greten, Jorge Machado

**Affiliations:** 1 ICBAS-UP—School of Medicine and Biomedical Sciences, University of Porto, 4050-313 Porto, Portugal; 2 CBSin—Center of BioSciences in Integrative Health, 4000-105 Porto, Portugal; 3 HSCM—Heidelberg School of Chinese Medicine, 69126 Heidelberg, Germany; 4 IPTC—Research Department in Complementary Medicine of the Portuguese Institute of Taiji and Qigong, 4470-765 Maia, Portugal; 5 LABIOMEP—Porto Biomechanics Laboratory, University of Porto, 4200-450 Porto, Portugal

**Keywords:** *Qigong*, students, reaction time, attention, learning capacity, academic stress, COVID-19

## Abstract

The recent COVID-19 pandemic has increased students’ stress as they may feel under increased pressure to have a good performance and compensate for the disruption to their education. Improving attention levels and learning capacity may assist in ameliorating academic performance. *Qigong* is a traditional Chinese medicine technique that appears to have positive effects on the management of mental health and may provide tools for coping with stressful situations. This paper explores data obtained while conducting a previous study and includes an excess of data from a total of 44 participants who were previously divided into an experimental *Qigong* group and a sham *Qigong* control group. The improvements in specific auditory processing and reaction times may indicate benefits in attention and learning capacity. These improvements were more pronounced in the experimental *Qigong* group compared to the sham *Qigong* group. *Qigong* may be able to assist in improving students’ academic performance and can be easily integrated into physical education classes. It could also assist students to cope with the increased academic pressure resulting from the COVID-19 pandemic context.

## 1. Introduction

In the last decades, there has been increasing concern about the mental health of children and young people [1,2,3,4]. It is usually considered that high academic pressure and the need to succeed in school can have a negative impact on the psychological well-being of students [5,6,7,8] and can even lead to the development of school-related burnout [8,9,10]. Considering the already damaging effects of that phenomenon, the recent COVID-19 pandemic might have exacerbated it, as its impact on schools and educational institutions was severe [11], affecting no less than 1.5 billion learners during COVID-19’s first wave [12]. This impact resulted in a significant disruption of the usual educational processes in terms of decreased engagement and class participation as well as decreased opportunities for social interaction [13]. As well, teachers experienced an additional workload and stress due to increased barriers to engaging with students, parents, and colleagues [13], ultimately resulting in worse outcomes for students [14].

It is crucial to understand that academic pressure and the resulting stress are associated with depression, anxiety, and substance use disorders [15], perpetuating a cycle of lower school performance and achievement [16,17]. According to Merriam-Webster’s Collegiate Dictionary [18], attention can be defined as “the act or state of applying the mind to something” or “a condition of readiness for such attention involving especially a selective narrowing or focusing of consciousness and receptivity”. Improved dimensions of conditioned reaction times seem to be related to improved attention as well as to a better learning capacity [19] and, thus, specifically in children and adolescents, school performance [20,21].

Considering all this, effective tools that assist in handling school pressures, especially in examination phases, must be considered and implemented in schools. Techniques that assist directly or indirectly in the improvement of reaction time and, therefore, attention and learning capacity must be carefully considered and studied.

*Qigong*, as a traditional Chinese medicine technique, incorporates breathing techniques with static or dynamic exercises and meditative processes to attain specific therapeutic results [22]. It may be considered an applied psychophysiological feedback technique that may be guided by the student himself, allowing him to learn how to control the body’s functions and processes, achieving homeostasis [22]. As well, the Heidelberg model of traditional Chinese medicine considers *Qigong* as a traditional vegetative biofeedback technique with many health-related applications [23,24].

This technique seems to have positive effects on the management of mental health and may provide tools to overcome stressful contexts [25,26,27,28,29]. As well, some cognitive and behavioural improvements seem to be produced by the practice of therapeutic *Qigong* [24,30,31].

Especially for students, some studies have shown that *Qigong* can be a useful tool to assist in handling stress, raising levels of motivation, modulating behaviour, and improving focus [22,32,33,34,35]. It is suggested that it can also be applied as a mindfulness-based cognitive behavioural therapy in schools [33]. This brief report aims to explore the data collected in a previous study and analyse the specific measures through which *Qigong* can promote academic performance in young students and help to offset the negative effects of COVID-19 in the school context.

## 2. Methodology

This paper examines data obtained while conducting a previous study [36]. The sample consisted of 66 adolescents attending the eighth grade. However, the data examined in this paper focuses on the experimental and placebo groups with a total of 44 participants.

The experimental group (54.5% ♀, 45.5% ♂, age range 12 to 14 years old, mean 12.95 years old) performed the Heidelberg model’s White Ball *Qigong* exercise as described by Greten [23], with a 5-min duration at the end of gym classes, twice a week for four weeks. The placebo group (50% ♀, 50% ♂, age range 12 to 14 years old, mean 13 years old) performed sham *Qigong* by adopting the White Ball exercise posture while watching television. Both groups were instructed to do the exercises daily at home.

The MP36 system (from Biopac Systems, Inc., Goleta, CA, USA) was used for data retrieval. The participants had to listen to three sequences of 10 sounds each and press a button upon hearing the stimulus.

The first and second sequences measured reaction time related to attention (random stimuli), while the third sequence measured reaction time specifically related to learning capacity (rhythmic stimuli). Data were collected at the beginning of the study (T0), after 2 weeks (T1), and at the beginning of the school assessment period after 4 weeks (T2).

## 3. Results and Discussion

**Figure 1:**
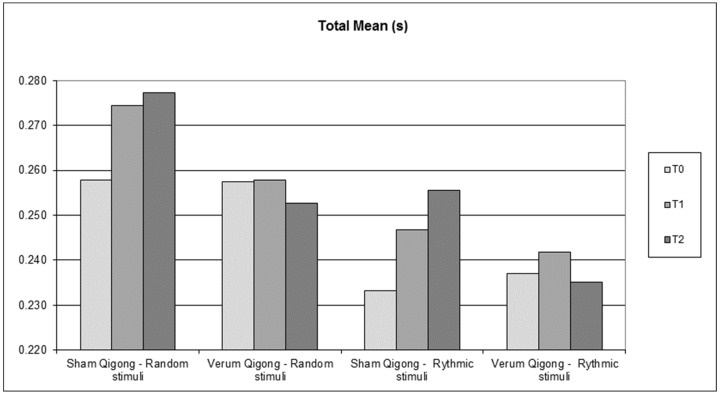
Stimuli mean results at each assessment time for both groups.

The results according to Figure 1 suggest that participants tended to demonstrate higher values of reaction time scores in T1 compared to T0. For both stimuli, the *true Qigong* group performed better, with smaller increases in reaction time. In T2, the tendency to increase reaction time remained in the sham *Qigong* group, while the experiemental *Qigong* group showed a decrease in reaction time for both stimuli.

**Figure 2:**
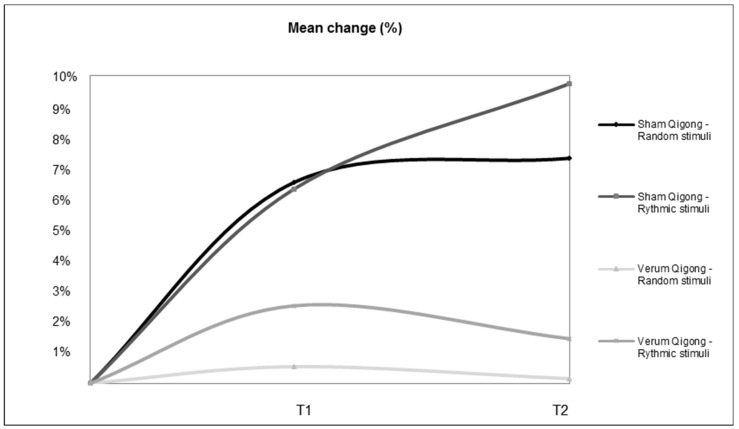
Mean percentage change in reaction time for both groups based on the type of stimuli.

The change can also be observed in Figure 2, which shows the development of each group according to the type of stimuli. Overall, the sham *Qigong* group showed increased reaction times, while the experimental *Qigong* group showed a tendency towards stabilisation and even some improvement in reaction times.

These data suggest that experimental *Qigong* may be able to stabilise or decrease reaction times. Improvements in these measures may be associated with improvements in attention (random stimuli) and learning capacity (rhythmic stimuli). In both the experimental and sham groups, the response to the random stimulus took longer. This may be due to the greater involvement of the brain (auditory and decision time) and spinal (final peripheral execution) procedures, while rhythmic stimulation is more related to spinal reflexes.

The timeframe in which this data was retrieved may also suggest that experimental *Qigong* can counteract anxiety’s destabilising effect, namely on neuromuscular reactivity.

For the same reason, as the school’s assessment period approached, the sham *Qigong* participants could not achieve such results, and their performance in the tests was progressively lower.

The previously published study [36] already showed positive effects on the attention levels of this sample by using the d2 test of attention assessment. In addition, another study [37], also using White Ball *Qigong* but with an older population, a showed positive effects on auditory attention mechanisms, as verified by the increasing speed of reaction times in the experimental group compared with a control group. The same outcome measures were applied (MP36 System by Biopac Systems), and the study also concluded that specific White Ball *Qigong* education (conditioning) may be important to maximise the beneficial effects of the technique. As well, meta-analyses on a different population indicate that these techniques may be beneficial in the improvement of global cognitive function, memory, learning, mental speed, attention, visuospatial perception, language, ideas, abstraction, and figural creation [30,31]. The effects can potentially be related to the regulation of the plasma brain-derived neurotrophic factor (BDNF), as suggested by the study of Lin, Cui, Yang, Yang, Feng, Wahner-Roedler, Zhou, Salinas, Mallory, Do, Bublitz, Chon, Tang, Bauer, and Xu [31]. BDNF is an essential molecule that is involved in plastic changes related to learning and memory [38]. As well, the pivotal role of BDNF in the potentiation of neuromuscular transmission [39] also supports our previously mentioned hypothesis regarding neuromuscular reactivity improvement.

According to our results and the exploration of this data, we propose that academic performance can be benefited by *Qigong* via the improvement of reaction times related to attention and learning capacity.

Overall, several mechanisms may be involved in the development of *Qigong*’s health-related benefits and the specific improvements in some cognitive functions [40,41,42,43]. These benefits may vary according to technique and population, and for that, systematic investigation in the field should be developed using standardised methods and well-developed studies. Despite no side effects being reported in our sample, the protocol study of Guo et al. [44] suggests that *Qigong* may have the risk of developing a wide range of side effects. However, more scientific research is needed to properly assess the real risks and understand their dimensions. For these reasons, we suggest that future studies should be conducted with larger samples, with well-designed allocation procedures, and explore control group possibilities through the design of extended comparisons with multiple interventions and sham interventions.

## 4. Conclusions

The results of this data analysis suggest that *Qigong* can be a useful tool for improving student performance and can be easily integrated into physical education classes. As such, it may also prove to be a useful tool in dealing with the increased pressures and demands that COVID-19 has caused in the school environment.

## Data Availability

The data presented in this study are available on request from the corresponding author. The data are not publicly available due to privacy restrictions.
